# Comprehensive behavioral analysis of the *Cdkl5* knockout mice revealed significant enhancement in anxiety- and fear-related behaviors and impairment in both acquisition and long-term retention of spatial reference memory

**DOI:** 10.1371/journal.pone.0196587

**Published:** 2018-04-27

**Authors:** Kosuke Okuda, Keizo Takao, Aya Watanabe, Tsuyoshi Miyakawa, Masashi Mizuguchi, Teruyuki Tanaka

**Affiliations:** 1 Department of Developmental Medical Sciences, Graduate School of Medicine, The University of Tokyo, Bunkyo-ku, Tokyo, Japan; 2 Section of Behavior Patterns, Center for Genetic Analysis of Behavior, National Institute for Physiological Sciences, Okazaki, Aichi, Japan; 3 Division of Systems Medical Science, Institute for Comprehensive Medical Science, Fujita Health University, Toyoake, Aichi, Japan; Technion Israel Institute of Technology, ISRAEL

## Abstract

Mutations in the *Cyclin-dependent kinase-like 5 (CDKL5)* gene cause severe neurodevelopmental disorders. Recently we have generated *Cdkl5* KO mice by targeting exon 2 on the C57BL/6N background, and demonstrated postsynaptic overaccumulation of GluN2B-containing *N*-methyl-*D*-aspartate (NMDA) receptors in the hippocampus. In the current study, we subjected the *Cdkl5* KO mice to a battery of comprehensive behavioral tests, aiming to reveal the effects of loss of CDKL5 in a whole perspective of motor, emotional, social, and cognition/memory functions, and to identify its undetermined roles. The neurological screen, rotarod, hot plate, prepulse inhibition, light/dark transition, open field, elevated plus maze, Porsolt forced swim, tail suspension, one-chamber and three-chamber social interaction, 24-h home cage monitoring, contextual and cued fear conditioning, Barnes maze, and T-maze tests were applied on adult *Cdkl5* -/Y and +/Y mice. *Cdkl5* -/Y mice showed a mild alteration in the gait. Analyses of emotional behaviors revealed significantly enhanced anxiety-like behaviors of *Cdkl5* -/Y mice. Depressive-like behaviors and social interaction of *Cdkl5* -/Y mice were uniquely altered. The contextual and cued fear conditioning of *Cdkl5* -/Y mice were comparable to control mice; however, *Cdkl5* -/Y mice showed a significantly increased freezing time and a significantly decreased distance traveled during the pretone period in the altered context. Both acquisition and long-term retention of spatial reference memory were significantly impaired. The morphometric analysis of hippocampal CA1 pyramidal neurons revealed impaired dendritic arborization and immature spine development in *Cdkl5* -/Y mice. These results indicate that CDKL5 plays significant roles in regulating emotional behaviors especially on anxiety- and fear-related responses, and in both acquisition and long-term retention of spatial reference memory, which suggests that focus and special attention should be paid to the specific mechanisms of these deficits in the CDKL5 deficiency disorder.

## Introduction

The *Cyclin-dependent kinase-like 5 (CDKL5)* gene (OMIM #300203) on the chromosome Xp22 region encodes for a serine/threonine kinase, CDKL5 [1]. Since 2003, mutations in the *CDKL5* gene have been identified in patients with severe neurodevelopmental disorders characterized by early-onset intractable epilepsy and severe psychomotor retardation [2–4]. The majority of patients with *CDKL5* mutations/deletions are heterozygous females, and are diagnosed as atypical Rett syndrome, West syndrome, or early infantile epileptic encephalopathy 2 (EIEE2) (OMIM #300672) [4]. Pathogenic mutations of *CDKL5* cause loss-of-function (LOF) of the kinase [5].

CDKL5 is expressed widely in most tissues, with highest levels in brain, thymus, and testis [6]. Within neurons, it localizes in nucleus, neurites, growth cones, dendritic spines, and at the postsynaptic density (PSD) of excitatory synapses [6–9]. Multiple localization of CDKL5 suggests its various roles in living organisms. Recent studies have revealed some of its molecular functions, such as the influence on RNA splicing activity by association with the nuclear speckle molecular machinery [10]; brain-derived neurotrophic factor (BDNF)-induced activation of Rho-GTPase Rac1, and regulation of neurite outgrowth [8]; the interaction with palmitoylated PSD-95, which regulates synaptic targeting of CDKL5 [11]; the interaction with Shootin 1 to regulate neuronal polarization [12]; and localization at the centrosome and midbody, which is required for faithful cell division [13]. We have recently shown that CDKL5 controls postsynaptic localization of GluN2B-containing *N*-methyl-*D*-aspartate (NMDA) receptors in the hippocampus, and regulates seizure susceptibility [9]. CDKL5 has been to shown to phosphorylate DNA methyltransferase 1 (DNMT1) [14]; amphiphysin 1 [15]; Netrin-G1 ligand (NGL-1) [7]; histone deacetylase 4 (HDAC4) [16]; and Methyl-CpG binding protein 2 (MeCP2), which in turn acts as a transcriptional repressor of *Cdkl5* [17, 18].

In order to elucidate the *in vivo* functions and the LOF mechanisms of CDKL5, three independent laboratories including us have generated the *Cdkl5* knockout (KO) mice, by targeting exon 6 [19, 20], exon 4 [21], or exon 2 (our mice) [9]. Molecular biological analyses of these mutant mice have revealed alterations in auditory-evoked event-related potentials (ERPs) and disruption of AKT-mammalian target of rapamycin (mTOR) signaling pathway [19]; altered neurogenesis and abnormal dendritic branching in the dentate gyrus, and disruption in AKT/glycogen synthase kinase-3β (GSK-3β) signaling pathway [22, 23]; abnormal electroencephalograph (EEG) responses to convulsant treatment, decreased visual evoked responses (VEPs), and alterations in the AKT/ribosomal protein S6 (RPS6) signaling pathway [21]; selective defect of GAD67 in the molecular layer, a higher reduction in spontaneous GABA efflux in the synaptosomes, and reduced BDNF expression in the cerebellum [24]; a severe reduction of c-Fos expression, increased VGluT1, downregulated PSD-95 and Homer, and higher density of parvalbumin-positive interneurons in the primary visual cortex [25]; and a significant reduction of spine density and PSD-95-positive synaptic puncta, and impaired long-term potentiation (LTP) in the somatosensory cortex [26]. We have identified significant hyperexcitability to NMDA, aberrant and excessive accumulation of GluN2B-containing NMDA receptors (NMDARs) at postsynapses, and enhanced NMDAR-mediated excitatory postsynaptic currents (EPSCs) and LTP in the hippocampus of *Cdkl5* KO mice [9].

Several studies have reported behavioral phenotypes of previously generated *Cdkl5* KO mice. Hyperactivity, impairment in coordination, sociability and memory, and autistic and ADHD-like behaviors have been identified in exon-6-targeted KO mice [19, 27]. Increased hindlimb clasping, decreased home cage activity, impairment in coordination and memory have been identified in exon-4-targeted KO mice [21–24]. Thus, a certain amount of similar phenotypes has been obtained in different laboratories using different mice and protocols. However, there is some variability in the results among studies, and certain domains of behaviors are not closely explored, especially on emotional responses. No conclusion has been reached as yet in regard to the overall neurological outcomes of the loss of CDKL5. Importantly, behavioral endophenotypes of mutant mice should be used as the key biomarkers to evaluate preclinical therapeutic trials. These emphasize the necessity of more thorough characterization of behaviors of *Cdkl5* KO mice.

In the current study, we aimed to reveal the behavioral effects of the loss of CDKL5 in a whole perspective of motor, emotional, social, and cognition/memory functions, and to identify its undetermined roles, by adopting a battery of comprehensive behavioral tests to our *Cdkl5* KO mice generated by targeting exon 2 on the C57BL/6N background, which are distinct from the previous mutant mice targeting exon 4 or 6 on the C57BL/6J background. We find that our *Cdkl5* KO mice exhibit significantly enhanced anxiety- and fear-related behaviors. Depressive-like behaviors and social interaction of *Cdkl5* KO mice are uniquely altered. Both acquisition and long-term retention of spatial reference memory are significantly impaired. These results indicate that CDKL5 plays significant roles in regulating emotional behaviors especially on anxiety- and fear-related responses, and in both acquisition and long-term retention of spatial reference memory, which suggests that focus and special attention should be paid to the specific mechanisms of these deficits in the CDKL5 deficiency disorder.

## Results

### *Cdkl5* -/Y mice show normal muscular strength and sensory activities

**Fig 1:**
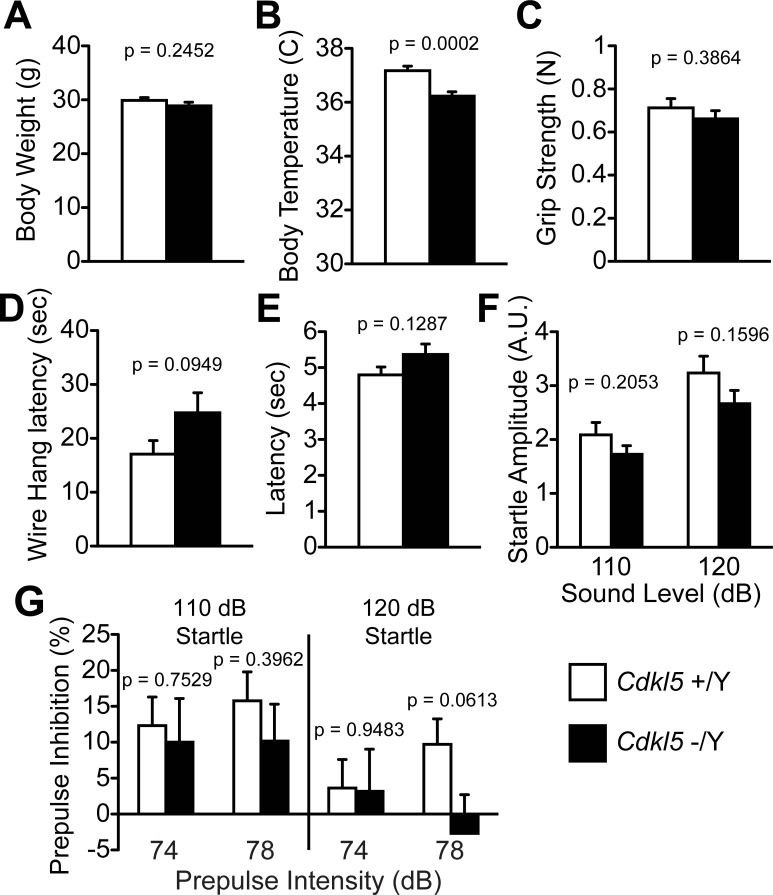
General health and neurological screening. (A) There is no significant difference in the body weight between *Cdkl5* +/Y and -/Y mice. (B) *Cdkl5* -/Y mice show a significantly lower body temperature than control mice. (C) There is no significant difference in the grip strength between two genotypes. (D) There is no significant difference in the wire hang latency between two genotypes. (E) There is no significant difference in the hot plate test latency between two genotypes. (F) Acoustic startle response. There is no significant difference between two genotypes either at 110dB or 120 dB sound level. (G) There are no significant differences in the prepulse inhibition (PPI) (%) between genotypes. In *Cdkl5* +/Y mice, the PPI induced by 78dB prepulse was larger than that of 74dB prepulse in both startle conditions; however in *Cdkl5* -/Y mice, 78dB prepulse does not induce larger PPI over 74dB prepulse in either startle condition, and the genotype difference of PPI induced by 78dB prepulse in 120dB startle condition is markedly large. *Cdkl5* +/Y, n = 22; *Cdkl5* -/Y, n = 22. Data indicate means ± SEM.

In order to reveal the neurological and behavioral roles of CDKL5, we subjected adult *Cdkl5* -/Y and +/Y mice to a comprehensive battery of behavioral tests (Table 1) [28]. In the general health check and neurological screening, *Cdkl5* -/Y mice showed no significant change in the whisker, coat, righting reflex, whisker-twitch, ear-twitch, reaching, body weight (Fig 1A), but showed a significantly lower rectal temperature compared to the control mice (F_1,42_ = 16.554, p = 0.0006, one-way ANOVA, Fig 1B). There were no significant differences between genotypes in the grip strength (Fig 1C), wire hang (Fig 1D), hot plate test (Fig 1E), and acoustic startle response (Fig 1F). The prepulse inhibition (PPI) tests were performed with four combinations of prepulse and startle stimulus; 74dB prepulse-110dB startle, 78dB prepulse-110dB startle, 74dB prepulse-120dB startle, and 78dB prepulse-120 dB startle. There were no significant differences of PPI (%) between genotypes in these combinations (Fig 1G). It is of note that in *Cdkl5* +/Y mice, the PPI induced by 78dB prepulse was larger than that of 74dB prepulse in both startle conditions; however in *Cdkl5* -/Y mice, 78dB prepulse did not induce larger PPI over 74dB prepulse in either startle condition, and the genotype difference of PPI induced by 78dB prepulse in 120dB startle condition was markedly large (F_1,42_ = 3.697, p = 0.0613, one-way ANOVA, Fig 1G). These results suggest an alteration of optimal prepulse intensities against each startle, or possible alteration of sensorimotor gating function in *Cdkl5* -/Y mice.

**Table 1:** Comprehensive battery of behavioral tests.

	Test	Measurement
1	General health/neurological screen	body weight, rectal temperature, whisker, coat, simple reflexes
2	Wire hang	muscle strength
3	Grip strength test	muscle strength
4	Gait analysis	gait, locomotion, motor function
5	Hot plate test	nociception
6	Rotarod	motor coordination
7	Startle response & prepulse inhibition	sensory-motor gating
8	Light/dark transition	anxiety-like behavior
9	Open Field	activity, anxiety-like behavior
10	Elevated plus maze	anxiety-like behavior
11	Social interaction (novel environment)	social behavior
12	Social interaction (Crawley)	social behavior
13	24-hr home cage monitoring	24-hr activity, social behavior
14	Porsolt Forced Swim	depression-like behavior
15	Tail suspension	depression-like behavior
16	Contextual and cued fear conditioning	context memory
17	Barnes maze	reference memory, preservation,
18	T-maze	working memory

### *Cdkl5* -/Y mice exhibit a mild alteration in the gait

**Fig 2:**
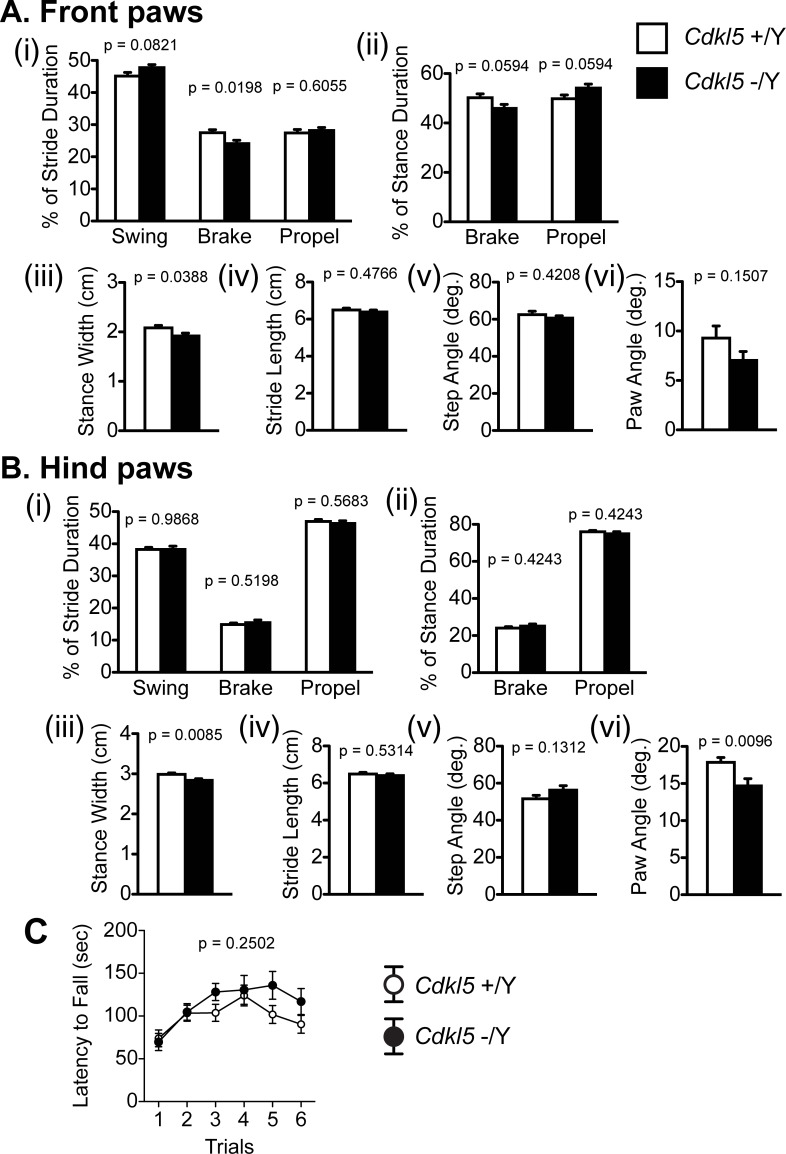
*Cdkl5* -/Y mice exhibit a mild alteration in the gait. (A) Gait analysis of front paws. *Cdkl5* +/Y, n = 22; *Cdkl5* -/Y, n = 19. (i) Percent of stride duration. *Cdkl5* KO mice show a significantly lower percentage of brake duration. (ii) Percent of stance duration. There is no significant difference in the duration of brake or propel between two genotypes. (iii) *Cdkl5* -/Y mice show a significantly shorter stance width. (iv) There is no significant difference in the stride length between two genotypes. (v) There is no significant difference in the step angle between two genotypes. (vi) There is no significant difference in the paw angle between two genotypes. (B) Gait analysis of hind paws. *Cdkl5* +/Y, n = 22; *Cdkl5* -/Y, n = 19. (i) There are no significant differences in the duration of swing, brake, or propel between two genotypes. (ii) There is no significant difference in the duration of brake or propel between two genotypes. (iii) *Cdkl5* -/Y mice show a significantly shorter stance width. (iv) There is no significant difference in the stride length between two genotypes. (v) There is no significant difference in the step angle between two genotypes. (vi) *Cdkl5* -/Y mice show a significantly small paw angle compared with the control. (C) The rotarod test. There is no significant difference in the latency to fall between two genotypes. *Cdkl5* +/Y, n = 22; *Cdkl5* -/Y, n = 22. Data indicate means ± SEM.

Previous studies reported impaired motor coordination of *Cdkl5* KO mice, using the rotarod and CatWalk tests [19, 24]. We also identified an altered gait of our *Cdkl5* -/Y mice. The stride pattern of the front paws (% of brake duration: F_1,38_ = 5.92, p = 0.0198, one-way ANOVA, Fig 2A(i)) and the angles of hind paws (F_1,38_ = 7.451, p = 0.0096, one-way ANOVA, Fig 2B(vi)) were significantly different, and the stance widths of both front and hind paws were significantly shorter (front paw: F_1,38_ = 4.58, p = 0.0388, one-way ANOVA, hind paw: F_1,38_ = 7.708, p = 0.0085, one-way ANOVA, Fig 2A(iii) and 2B(iii)). These results suggest a mild coordination disturbance in our *Cdkl5* KO mice. The one-day rotarod test showed no significant difference in the latency to fall between genotypes (F_1,42_ = 1.36, p = 0.2502, one-way ANOVA, Fig 2C).

### *Cdkl5* -/Y mice show significantly enhanced anxiety-like behaviors

**Fig 3:**
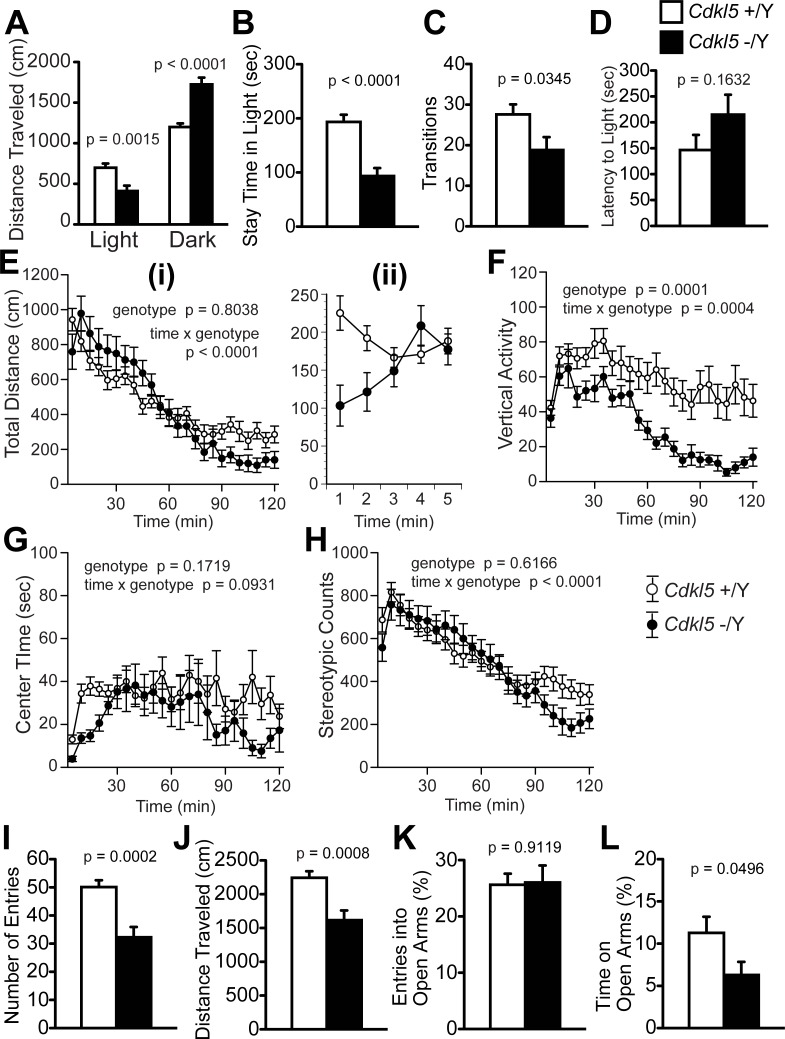
*Cdkl5* -/Y mice show significantly enhanced anxiety-like behaviors. (A-D) The light/dark transition test. (A) *Cdkl5* -/Y mice show a significantly decreased distance traveled in the light chamber and an increased distance in the dark chamber. (B) *Cdkl5* -/Y mice show a significantly decreased stay time in the light chamber. (C) *Cdkl5* -/Y mice show a significantly decreased number of transitions between chambers. (D) *Cdkl5* -/Y mice show a tendency to increase in the latency to light chamber, but without statistical significance. (E-H) The open field test. (E) (i) Total distance traveled (cm) in 120 minutes. Each bin represents a mean value in 5 minutes. There is no significant difference between two genotypes (p = 0.8038). (ii) Total distance traveled during the first 5 minutes. Each bin represents a mean value in one minute. Marked reduction in the distance traveled of *Cdkl5* -/Y mice is observed. (F) The vertical activity. *Cdkl5* -/Y mice show a significantly decreased vertical activity in the whole period, compared with the control mice (p = 0.0001). There is a significant time x genotype effect (p = 0.0004). (G) Center time (sec). In the whole period, there is no significant difference of the center time between two genotypes (p = 0.1719). However, in the first 5 minutes (first bin), *Cdkl5* -/Y mice show a marked decrease in the center time. (H) Stereotypic counts. In the whole period, there is no significant difference between two genotypes (p = 0.1719), but time x genotype effect is significant (p < 0.0001). (I-L) The elevated plus maze test. (I) *Cdkl5* -/Y mice show a significantly decreased number of entries into all arms. (J) *Cdkl5* -/Y mice show a significantly decreased distance traveled. (K) There is no difference in the number of entries into open arms between two genotypes. (L) *Cdkl5* -/Y mice show a significantly decreased time spent on the open arms. *Cdkl5* +/Y, n = 22; *Cdkl5* -/Y, n = 22. Data indicate means ± SEM.

We examined anxiety-related behaviors of *Cdkl5* -/Y mice by the light/dark transition (Fig 3A–3D), open field (Fig 3E–3H), and elevated plus maze tests (Fig 3I–3L) [29]. In the light/dark transition test, *Cdkl5* -/Y mice showed a significantly decreased distance traveled in the light chamber (F_1,42_ = 11.464, p = 0.0015, one-way ANOVA, Fig 3A), an increased distance traveled in the dark chamber (F_1,42_ = 30.549, p < 0.0001, one-way ANOVA, Fig 3A), a decreased time to stay in the light chamber (F_1,42_ = 25.586, p < 0.0001, one-way ANOVA, Fig 3B), a decreased number of transition between chambers (F_1,42_ = 4.776, p = 0.0345, one-way ANOVA, Fig 3C), and a tendency to increase in the latency to light chamber (F_1,42_ = 2.014, p = 0.1632, one-way ANOVA, Fig 3D). These results of *Cdkl5* -/Y mice indicate inhibited exploration in the brightly lit, unprotected area, and suggest an enhancement of anxiety-like behaviors.

We performed the open field test for total duration of 120 minutes. In this test, exploration of a brightly lit, novel and unprotected environment is assessed during the first 5 minutes, and general activities during habituation to novelty are scored across a longer time [30]. In the whole period, there was no significant difference of total distance traveled between two genotypes (F_1,42_ = 0.063, p = 0.8038, two-way repeated measures ANOVA, Fig 3E(i)); however, during the first 5 minutes, *Cdkl5* -/Y mice showed a marked decrease of the traveled distance compared to control mice (Fig 3E(ii)). The vertical activity of *Cdkl5* -/Y mice was significantly decreased compared to control mice (F_1,42_ = 17.73, p = 0.0001, two-way repeated measures ANOVA, Fig 3F). The center time of *Cdkl5* -/Y mice in the whole period was not significantly different from that of control mice (F_1,42_ = 1.932, p = 0.1719, two-way repeated measures ANOVA, Fig 3G). However, *Cdkl5* -/Y mice showed a marked decrease in the center time during the first 5 minutes (see the first bin in Fig 3G). There was no significant genotype difference of total stereotypic counts in the whole period (F_1,42_ = 0.254, p = 0.6166, two-way repeated measures ANOVA, Fig 3H), but time x genotype effect was significant (F_23,966_ = 3.575, p < 0.0001, two-way repeated measures ANOVA, Fig 3H). The marked decrease in the traveled distance and center time during the first 5 minutes indicates a reduction of exploration in novel, unprotected environment, suggesting an enhancement of anxiety-like behaviors of *Cdkl5* KO mice. They also showed significantly different time x genotype effects on the distance traveled (Fig 3E(i)), vertical activity (Fig 3F) and stereotypic counts (Fig 3H), indicating altered emotional response during the period of habituation to novelty.

In the elevated plus maze test, *Cdkl5* -/Y mice showed significant decreases in the number of entries into all arms (F_1,42_ = 16.589, p = 0.0002, one-way ANOVA, Fig 3I) and distance traveled (F_1,42_ = 13.12, p = 0.0008, one-way ANOVA, Fig 3J). The open arm entries (%) of *Cdkl5* -/Y mice was not significantly different from that of control mice (F_1,42_ = 0.012, p = 0.9119, one-way ANOVA, Fig 3K). However, the time spent on the open arms was significantly decreased in *Cdkl5* -/Y mice (F_1,42_ = 4.086, p = 0.0496, one-way ANOVA, Fig 3L). The decrease in the number of entries and traveled distance indicates a reduction of locomotor activities of the KO mice. The percentage of open arm entries and that of time spent on open arms are parameters that reflect anxiety-related behaviors. *Cdkl5* KO mice showed a significant decrease in the open arm time (Fig 3L), but not in the open arm entries (Fig 3K). This may be because the percentage of open arm entries is less sensitive to anxiogenic/anxiolytic effects than that of time spent on the open arms [31]. The significant decrease in the time spent on the open arms indicates avoidance from unprotected environment, which suggests an enhanced anxiety-like behavior. Thus, all data from light/dark, open field, and elevated plus maze tests consistently indicate significantly enhanced anxiety-like behaviors of *Cdkl5* KO mice.

**Fig 4:**
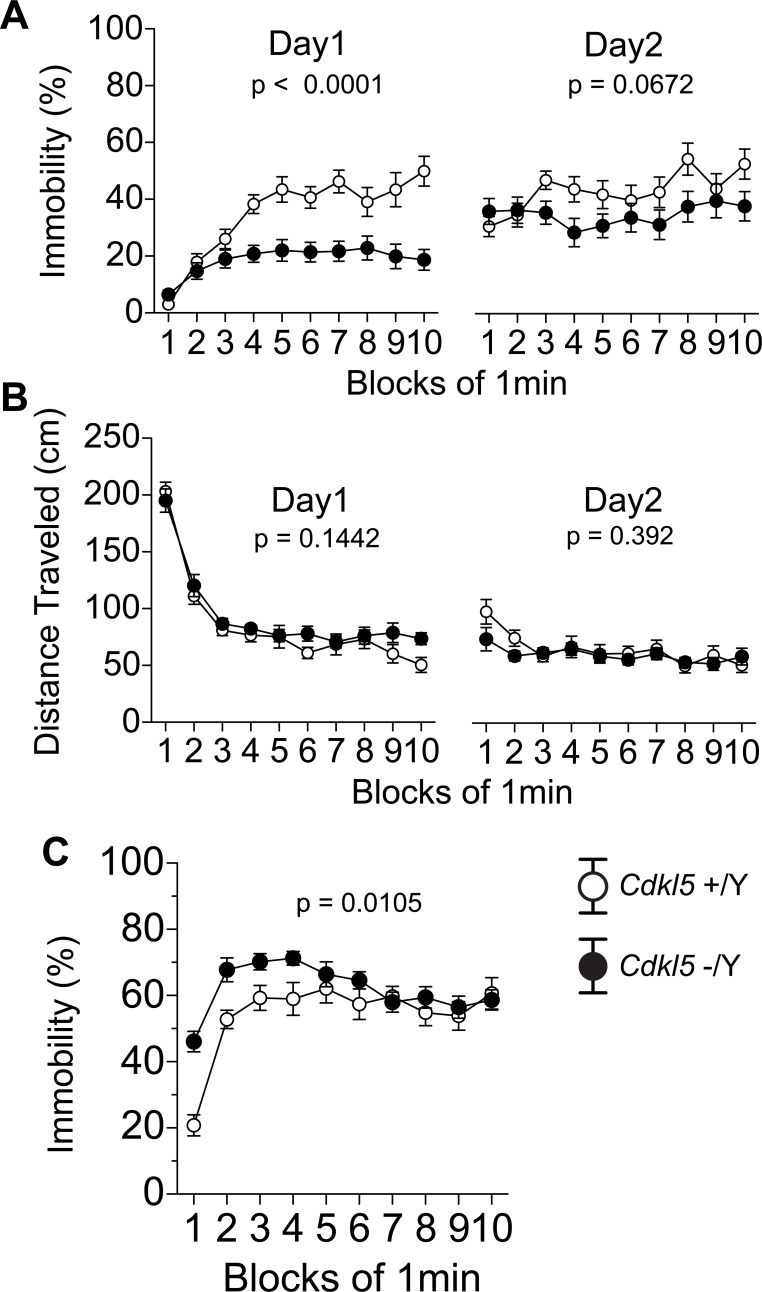
*Cdkl5* -/Y mice show altered depressive-like behaviors. (A, B) The Porsolt forced swim test. (A) *Cdkl5* -/Y mice show a significant decrease of immobility time in the first day (p < 0.0001), and a tendency to decrease in the second day (p = 0.0672). (B) There is no significant difference in the distance traveled between two genotypes either in day 1 or day 2. (C) The tail suspension test. *Cdkl5* -/Y mice show a significant increase of the immobility time (p = 0.0105). *Cdkl5* +/Y, n = 22; *Cdkl5* -/Y, n = 22. Data indicate means ± SEM.

To evaluate the depressive-like behaviors of *Cdkl5* -/Y mice, we applied the Porsolt forced swim test (Fig 4A and 4B) and the tail suspension test (Fig 4C). In the Porsolt forced swim test, the immobility time (%) and distance traveled (cm) were scored in each minute. The immobility time of *Cdkl5* -/Y mice was significantly decreased in the first day (F_1,42_ = 30.568, p < 0.0001, two-way repeated measures ANOVA, Fig 4A), and tended to decrease in the second day (F_1,42_ = 3.531, p = 0.0672, two-way repeated measures ANOVA, Fig 4A). The decreased immobility of *Cdkl5* -/Y mice indicates an anti-depressive-like behavior. The distance traveled did not significantly differ between genotypes (day 1, F_1,42_ = 2.214, p = 0.1442; day 2, F_1,42_ = 0.748, p = 0.392, two-way repeated measures ANOVA, Fig 4B). On the other hand, in the tail suspension test, *Cdkl5* -/Y mice showed a significant increase of the immobility time (F_1,42_ = 7.167, p = 0.0105, two-way repeated measures ANOVA, Fig 4C), indicative of a depressive-like behavior. The discrepancy between these two tests indicates that other factors may modulate the depressive-like responses of *Cdkl5* KO mice. In the forced swim test, the decreased immobility (Fig 4A) of KO mice was not accompanied by effective lateral movement (Fig 4B), which may suggest more panic-like response rather than the appropriate escape response. In the tail suspension test, the immobility % of KO mice seemed markedly increased during the former half of the test, peaking at the 4-minute time point; however, it decreased to the control level during the latter half. This time course suggests that the increased immobility during the former half may reflect excessive fear and anxiety in the aversive situation, and the gradual decrease may reflect habituation to the novel environment in *Cdkl5* KO mice. In these two tests, significantly increased anxiety of *Cdkl5* KO mice may be modulating the depressive-like responses, and may alter their behaviors.

### Social interaction of *Cdkl5* -/Y mice is uniquely altered

**Fig 5:**
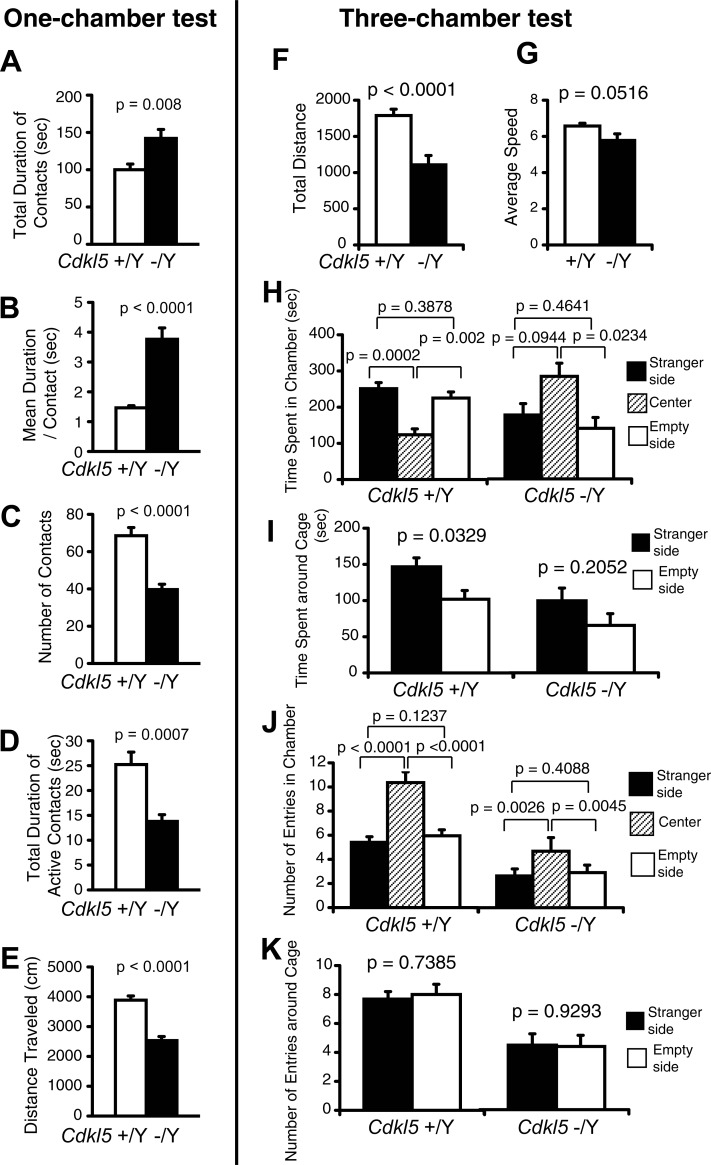
Social interaction of *Cdkl5* -/Y mice is uniquely altered. (A-E) The social interaction test in a novel environment (one-chamber test). *Cdkl5* -/Y mice show a significant increase in the total duration of contacts (A), a significant increase in the mean duration per contact (B), a significant decrease in the number of contacts (C), a significant decrease in the total duration of active contacts (D), and a significant decrease in the distance traveled (E). *Cdkl5* +/Y, n = 11 pairs; *Cdkl5* -/Y, n = 11 pairs. Data indicate means ± SEM. (F-K) The three-chamber social interaction test. *Cdkl5* -/Y mice show a significant decrease in the total distance traveled (F), and a tendency toward decrease in the average speed (G). (H) Time spent in each chamber (sec). *Cdkl5* -/Y mice show a significant preference for the center chamber, whereas *Cdkl5* +/Y mice spend significantly more time in other chambers. (I) Time spent around cage (sec). *Cdkl5* +/Y mice spend significantly more time around the mouse cage compared to the empty cage. *Cdkl5* -/Y mice showed a tendency toward preference for the mouse cage without statistical significance. (J) Number of entries in each chamber. Both genotypes similarly show a significant difference between the center and stranger chamber, a significant difference between the center and empty chamber, and no significant difference between the stranger and empty chamber. (K) Number of entries around cage. Both genotypes show no significant difference in the number of entries around cage between the stranger side and the empty side. *Cdkl5* +/Y, n = 22; *Cdkl5* -/Y, n = 22. Data indicate means ± SEM.

**Fig 6:**
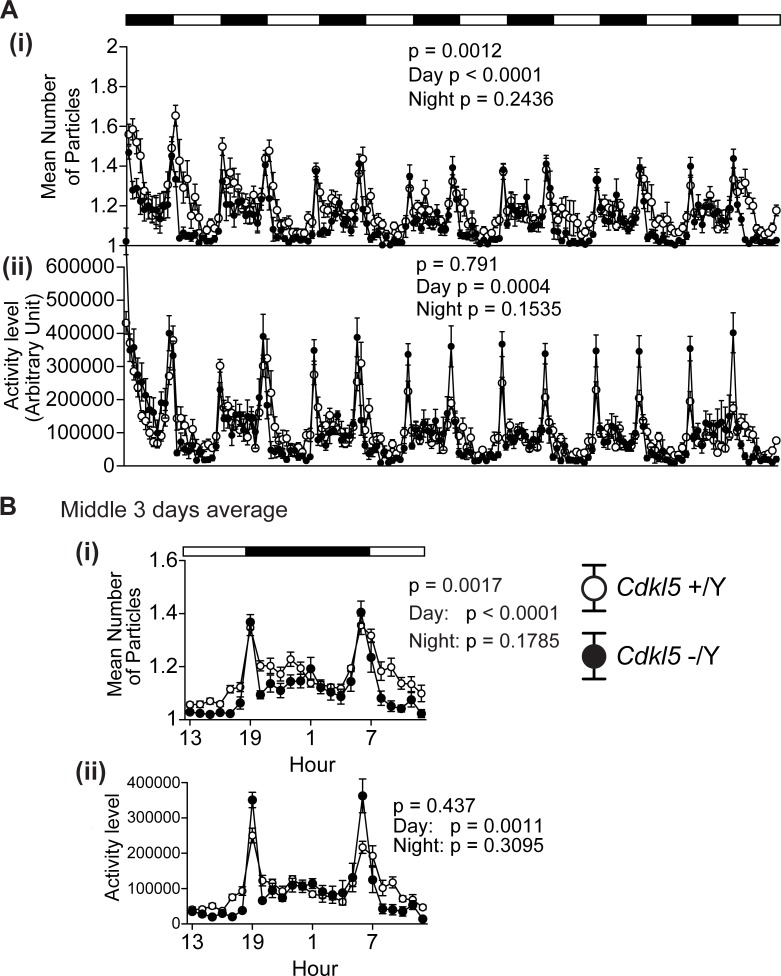
Twenty-four hour monitoring revealed increased interaction and decreased activity during daytime in *Cdkl5* -/Y mice. (A) (i) Mean number of particles. *Cdkl5* -/Y mice spend significantly more time staying together compared to *Cdkl5* +/Y mice during daytime (p < 0.001), but not in the nighttime (p = 0.2436). The genotype difference during the whole period was significant (p = 0.0012). (ii) Activity level (A.U.). The activity level of *Cdkl5* -/Y mice is significantly decreased compared to *Cdkl5* +/Y mice during daytime (p = 0.0004), but not in the nighttime (p = 0.1535). The genotype difference during the whole period was not significant (p = 0.791). (B) Mean values in the middle three days. *Cdkl5* -/Y mice show significant decreases in the number of particles (i) and activity level (ii) during daytime. *Cdkl5* +/Y, n = 11 pairs; *Cdkl5* -/Y, n = 11 pairs. Data indicate means ± SEM.

In order to evaluate the social interaction of *Cdkl5* -/Y mice, we conducted the social interaction test in a novel environment (one-chamber test) (Fig 5A–5E), three-chamber test (Fig 5F–5K), and 24-hour home cage monitoring (Fig 6). In the one-chamber test, we measured the total duration of contacts (Fig 5A), mean duration per contact (Fig 5B), number of contacts (Fig 5C), total duration of active contacts (Fig 5D), and distance traveled (Fig 5E). Active contacts denote physical contacts of two mice that run into each other and continue the locomotion for longer than 2 cm. Whereas physical contacts that reflect the social interaction correspond to spontaneous touching, rubbing, or sniffing behaviors toward each other, active contacts can occur just upon chance encounters during locomotion. The duration of active contacts should be greatly influenced by the activity level of the mice, and can be viewed as a parameter to reflect their activity. *Cdkl5* -/Y mice showed a significant increase in the total duration of contacts (F_1,20_ = 8.691, p = 0.008, one-way ANOVA, Fig 5A) and mean duration per contact (F_1,20_ = 35.588, p < 0.0001, one-way ANOVA, Fig 5B), whereas they showed a significant decrease in the number of contacts (F_1,20_ = 30.388, p < 0.0001, one-way ANOVA, Fig 5C), total duration of active contacts (F_1,20_ = 16.053, p = 0.0007, one-way ANOVA, Fig 5D), and distance traveled (F_1,20_ = 47.814, p < 0.0001, one-way ANOVA, Fig 5E). The significant increases in the total duration of contacts (Fig 5A) and the mean duration per contact (Fig 5B) of *Cdkl5* -/Y mice indicate a significantly increased social interaction between these mice. The decrease in the distance traveled (Fig 5E) of *Cdkl5* -/Y mice indicates hypoactivity possibly due to the enhanced anxiety of these mice in a novel environment, which is consistent with the open field test result (Fig 3E(ii)). Then, the decreases in the number of contacts (Fig 5C) and total duration of active contacts (Fig 5D) of *Cdkl5* -/Y mice should reflect the effect of hypoactivity in these mice. Thus these results demonstrate that when a *Cdkl5* KO mouse is exposed to an unfamiliar mouse in a novel environment, the mice avoid exploring and preferred staying with each other. These behavioral phenotypes should represent the combined effects of the anxiety-like behaviors and sociability per se. In *Cdkl5* KO mice, the sum of the anxiety in a novel environment and their sociability should exceed the anxiety to an unfamiliar mouse, and drive them to stay together. The genotype effect on sociability per se could not be determined by this test.

In the three-chamber social interaction test, the preference of the mouse can be quantified based on the time spent around a wire cage containing a stranger mouse vs. an empty cage [32]. In concordance with the one-chamber test, *Cdkl5* -/Y mice showed a significantly decreased distance traveled (F_1,42_ = 19.133, p < 0.0001, one-way ANOVA, Fig 5F). *Cdkl5* +/Y mice spent a significantly shorter time in the center chamber than in other chambers (time spent in chamber: center vs. stranger p = 0.0002; center vs. empty p = 0.002, paired t-test, Fig 5H), in contrast, *Cdkl5* -/Y mice spent a significantly longer time in the center chamber (time spent in chamber: center vs. stranger p = 0.0944; center vs. empty p = 0.0234, paired t-test, Fig 5H). These behaviors of *Cdkl5* -/Y mice indicate hypoactivity that should be derived from the enhanced anxiety in a novel environment. *Cdkl5* +/Y mice spent significantly more time around the stranger mouse cage compared to the empty cage (p = 0.0329, paired t-test, Fig 5I). *Cdkl5* -/Y mice also spent more time around the stranger mouse cage compared to the empty cage, but the difference did not reach statistical significance (p = 0.2052, paired t-test, Fig 5I). As to the number of entries in each chamber, both genotypes similarly showed a significant difference between the center and stranger chamber, a significant difference between the center and empty chamber, and no significant difference between the stranger and empty chamber (Fig 5J). Likewise, both genotypes showed no significant difference in the number of entries around cage between the stranger and empty side (Fig 5K). Thus, the three-chamber test results of *Cdkl5* -/Y mice demonstrate the significant hypoactivity but otherwise almost comparable social interaction to control mice.

Twenty-four hour monitoring in the home cage under familiar conditions was conducted for one week to analyze the interaction and circadian activities of *Cdkl5* KO mice. Social interaction was measured by counting the number of “particle”, which represents an image of each mouse (see Methods); two particles indicated that the mice were not in contact with each other, and one particle indicated contact between them. *Cdkl5* -/Y mice spent significantly more time staying together compared to control mice during daytime (mean number of particles F_1,20_ = 75.79, p < 0.0001, two-way repeated measures ANOVA, Fig 6A(i)), but not in the nighttime (mean number of particles F_1,20_ = 1.444, p = 0.2436, two-way repeated measures ANOVA, Fig 6A(i)). The genotype difference during the whole period was significant (F_1,20_ = 14.166, p = 0.0012, two-way repeated measures ANOVA, Fig 6A(i)). The activity level of *Cdkl5* -/Y mice was significantly decreased compared to control mice during daytime (F_1,20_ = 18.252, p = 0.0004, two-way repeated measures ANOVA, Fig 6A(ii)), but not in the nighttime (F_1,20_ = 2.201, p = 0.1535, two-way repeated measures ANOVA, Fig 6A(ii)). The mean values of those recorded in the middle three days clearly showed a significant decrease in the number of particles (F_1,20_ = 13.185, p < 0.0001, two-way repeated measures ANOVA, Fig 6B(i)) and activity level (F_1,20_ = 14.48, p = 0.0011, two-way repeated measures ANOVA, Fig 6B(ii)) of *Cdkl5* -/Y mice during daytime, indicating a significant increase of interaction and a significant decrease of locomotor activity. The behavioral outcomes of *Cdkl5* -/Y mice in the twenty-four hour home cage monitoring almost precisely mirror those in the one-chamber test; they show significantly reduced locomotor activity in aversive situations such as novel environment or daytime period, and a preference to stay with the other mouse. These altered social interactions of *Cdkl5* -/Y mice should be the consequence of the interaction of significantly enhanced anxiety with their sociability.

### *Cdkl5* -/Y mice show an increased freezing time and a decreased distance traveled during the pretone period in the altered context

**Fig 7:**
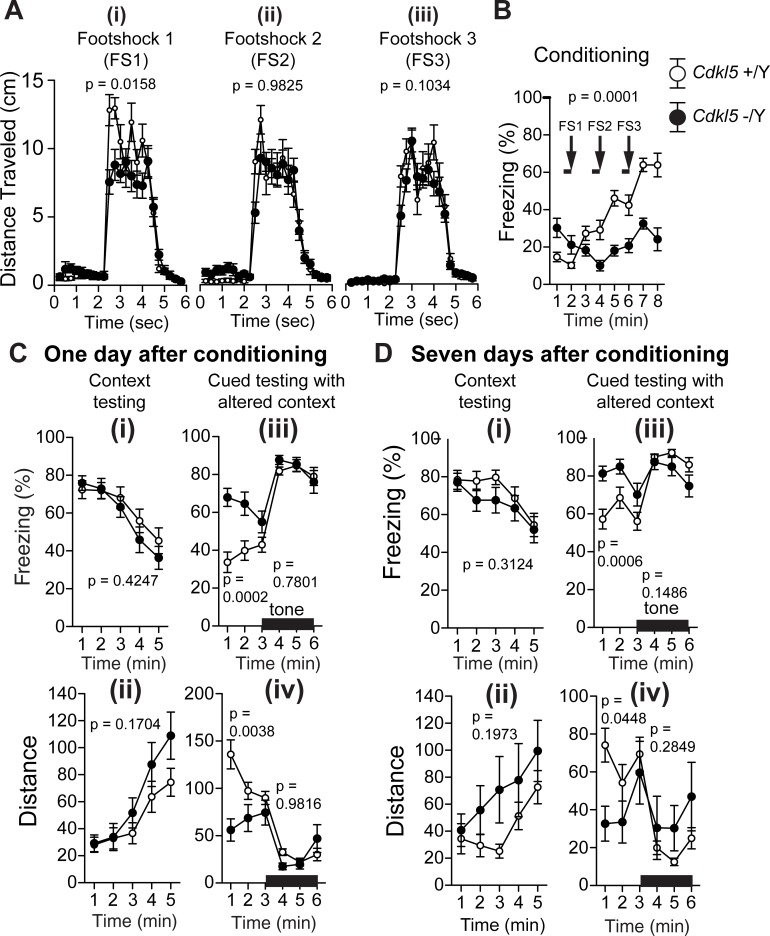
*Cdkl5* -/Y mice show an increased freezing time and a decreased distance traveled during the pretone period in the altered context. (A) Distance traveled (cm) at footshock 1 (FS1) (i), footshock 2 (FS2) (ii), and footshock 3 (FS3) (iii) during conditioning. *Cdkl5* -/Y mice show a significantly decreased distance traveled in response to the first footshock (p = 0.0158) compared with control mice. There are no significant differences upon the second and third footshock between two genotypes. (B) *Cdkl5* -/Y mice show a significantly decreased freezing time during conditioning. (C) (i,ii) Contextual testing one day after conditioning. There are no significant differences in the freezing time (i) and distance traveled (ii) between two genotypes. (iii,iv) Cued testing with altered context one day after conditioning. *Cdkl5* -/Y mice show a significantly increased freezing time (p = 0.0002) (iii) and a significantly decreased distance traveled (p = 0.0038) (iv) during the pretone period. There are no significant differences in the freezing time (iii) and distance traveled (iv) in response to the conditioned tone. (D) (i,ii) Contextual testing seven days after conditioning. There are no significant differences in the freezing time (i) and distance traveled (ii) between two genotypes. (iii,iv) Cued testing with altered context seven days after conditioning. *Cdkl5* -/Y mice show a significantly increased freezing time (p = 0.0006) (iii) and a significantly decreased distance traveled (p = 0.0448) (iv) during the pretone period. There are no significant differences in the freezing time (iii) and distance traveled (iv) in response to the conditioned tone. *Cdkl5* +/Y, n = 22; *Cdkl5* -/Y, n = 22. Data indicate means ± SEM.

*Cdkl5* -/Y mice were assessed for contextual and cued fear conditioning on one day and seven days after exposure to footshocks paired with auditory-conditioned stimuli. To examine shock sensitivity, the distance traveled was measured when the footshock was delivered during conditioning. Although both genotypes responded to footshocks, *Cdkl5* -/Y mice showed a significantly decreased distance traveled in response to the first footshock (F_1,42_ = 6.324, p = 0.0158, two-way repeated measures ANOVA, Fig 7A(i)), but not upon the second (F_1,42_ = 0.000488, p = 0.9825, two-way repeated measures ANOVA, Fig 7A(ii)) and third footshock (F_1,42_ = 2.772, p = 0.1034, two-way repeated measures ANOVA, Fig 7A(iii)). In the conditioning phase, control mice showed a stepwise increase in the freezing time upon each footshock; however, *Cdkl5* -/Y mice did not show an increase in the freezing time in response to successive footshocks (genotype effect: F_1,42_ = 18.023, p = 0.0001, two-way repeated measures ANOVA, Fig 7B). In the contextual test on one day after conditioning (Fig 7C), there were no significant differences between genotypes in the freezing time (F_1,42_ = 0.65, p = 0.4247, two-way repeated measures ANOVA, Fig 7C(i)) and distance traveled (F_1,42_ = 1.945, p = 0.1704, two-way repeated measures ANOVA, Fig 7C(ii)). However, in the altered context, *Cdkl5* -/Y mice showed a significantly increased freezing time (F_1,42_ = 16.869, p = 0.0002, two-way repeated measures ANOVA, Fig 7C(iii)) and a significantly decreased distance traveled (F_1,42_ = 9.39, p = 0.0038, two-way repeated measures ANOVA, Fig 7C(iv)) compared with control mice during the pretone period, whereas there were no significant genotype differences of the percent time freezing (F_1,42_ = 0.079, p = 0.7801, two-way repeated measures ANOVA, Fig 7C(iii)) and distance traveled (F_1,42_ = 0.001, p = 0.9816, two-way repeated measures ANOVA, Fig 7C(iv)) in response to the conditioned tone. The tests on seven days after conditioning yielded almost same results as the previous tests, i.e. no significant genotype differences of the percent time freezing (F_1,42_ = 1.046, p = 0.3124, two-way repeated measures ANOVA, Fig 7D(i)) and distance traveled (F_1,42_ = 1.716, p = 0.1973, two-way repeated measures ANOVA, Fig 7D(ii)) in the contextual test, a significantly increased freezing time (F_1,42_ = 13.699, p = 0.0006, two-way repeated measures ANOVA, Fig 7D(iii)) and a significantly decreased distance traveled of *Cdkl5* -/Y mice (F_1,42_ = 4.278, p = 0.0448, two-way repeated measures ANOVA, Fig 7D(iv)) in the altered context during the pretone period, and no significant genotype differences of freezing (F_1,42_ = 2.166, p = 0.1486, two-way repeated measures ANOVA, Fig 7D(iii)) and distance traveled (F_1,42_ = 1.173, p = 0.2849, two-way repeated measures ANOVA, Fig 7D(iv)) in the altered context with cued tone. Thus, these results demonstrate that whereas the contextual and cued fear conditioning are comparable to control mice, *Cdkl5* KO mice show a significantly increased freezing time and a significantly decreased distance traveled during the pretone period in the altered context, both on one day and seven days after conditioning.

### Both acquisition and long-term retention of spatial reference memory are significantly impaired in *Cdkl5* -/Y mice

**Fig 8:**
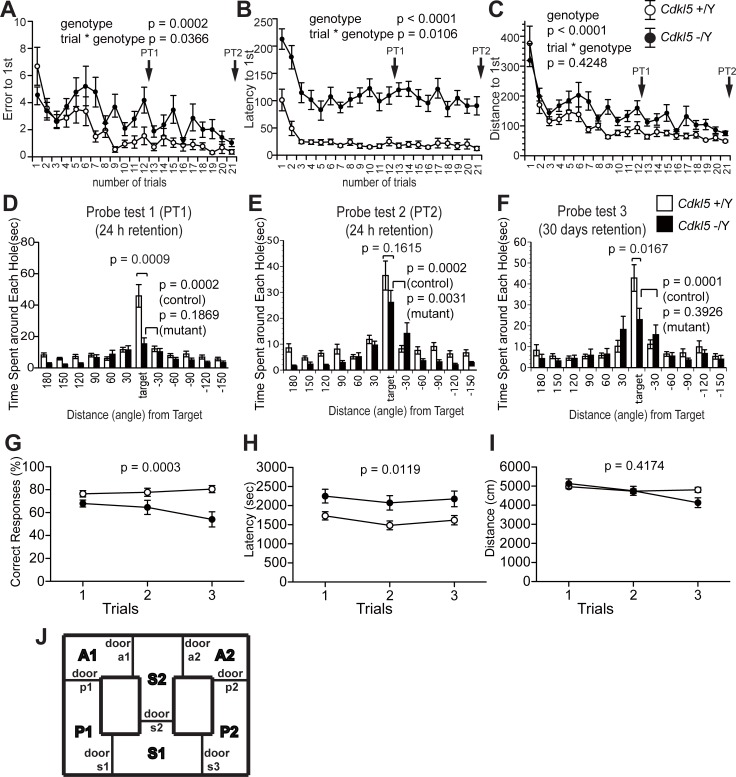
Acquisition and long-term retention of reference memory, and working memory are significantly impaired in *Cdkl5* -/Y mice. (A-F) Barnes maze test. Number of errors (A), latency (B), distance traveled (C) to reach the target during training. *Cdkl5* -/Y mice show a significant increase in the number of errors (p = 0.0002) (A), latency (p < 0.0001) (B) and distance traveled (p < 0.0001) (C) to reach the target hole, compared to the control mice. (D) In the first probe test (PT1) performed 24 hours after the 12th training, *Cdkl5* -/Y mice spend significantly less time at the target hole than control mice (p = 0.0009), and spend similar time at the adjacent hole to the target hole (p = 0.1869). (E) In the second probe test (PT2) performed 24 hours after the 21st training, *Cdkl5* -/Y mice spend similar time at the target hole to the control mice (p = 0.1615), and spend significantly more time at the target hole compared to the adjacent hole (p = 0.0031). (F) In the third probe test after 30 days, *Cdkl5* -/Y mice spend significantly less time at the target hole than control mice (p = 0.0167), and spend similar time at the adjacent hole to the target hole (p = 0.3926). (G-J) T-maze test. (G) *Cdkl5* -/Y mice show a significantly decreased percentage of correct responses than control mice in all 3 trials. (H) *Cdkl5* -/Y mice spend significantly longer time to finish 10 trials than control mice. (I) There is no significant difference in the distance traveled between two genotypes. (J) Scheme of the T-maze. *Cdkl5* +/Y, n = 22; *Cdkl5* -/Y, n = 22. Data indicate means ± SEM.

Fuchs et al. reported impaired spatial memory in the *Cdkl5* exon-4-targeted KO mice by the Morris water maze test [23]. We applied the Barnes maze test to analyze spatial memory acquisition and long-term retention of our *Cdkl5* KO mice. During the training period, *Cdkl5* -/Y mice showed a significant increase in the number of error (genotype effect: F_1,42_ = 16.205, p = 0.0002, two-way repeated measures ANOVA, Fig 8A), latency (genotype effect: F_1,42_ = 49.822, p < 0.0001, two-way repeated measures ANOVA, Fig 8B) and distance (genotype effect: F_1,42_ = 22.602, p < 0.0001, two-way repeated measures ANOVA, Fig 8C) to reach the target hole, compared to the control mice. In the first probe test (PT1) performed 24 hours after the 12th training, *Cdkl5* -/Y mice spent significantly less time at the target hole than control mice (p = 0.0009, one-way ANOVA, Fig 8D), and spent similar time at the adjacent hole to the target hole (p = 0.1869, paired t-test, Fig 8D), indicating the impaired acquisition of spatial reference memory. We performed additional 9 training trials until *Cdkl5* -/Y mice learned the target site (overtraining), and these enabled *Cdkl5* -/Y mice to remember the target hole, as indicated by a progressive reduction in the number of errors (Fig 8A). In the second probe test (PT2) performed 24 hours after the 21st training, *Cdkl5* -/Y mice spent similar time at the target hole to the control mice (p = 0.1615, one-way ANOVA, Fig 8E), and spent significantly more time at the target hole compared to the adjacent hole (p = 0.0031, paired t-test, Fig 8E), indicating the acquisition of the spatial memory. In order to assess the long-term retention of the spatial memory, we performed the third probe test (PT3) after 30 days. Then, control mice spent significantly more time at the target hole compared to the adjacent hole (p = 0.0001, paired t-test, Fig 8F) indicating retention of the spatial memory; however, *Cdkl5* -/Y mice spent similar time at the adjacent hole to the target hole (p = 0.3926, paired t-test, Fig 8F), and significantly less time at the target hole compared to the control mice (p = 0.0167, one-way ANOVA, Fig 8F). Thus these results indicate that both acquisition and long-term retention of spatial reference memory are significantly impaired in *Cdkl5* KO mice.

### Working memory is impaired in *Cdkl5* -/Y mice

Fuchs et al. reported impaired working memory in the *Cdkl5* exon-4-targeted KO mice by the Y-maze test [22]. We applied the T-maze test to measure working memory of our *Cdkl5* KO mice. *Cdkl5* -/Y mice showed a significantly decreased percentage of correct responses than control mice in all 3 trials (F_1,42_ = 15.498, p = 0.0003, two-way repeated measures ANOVA, Fig 8G). Twelve mice out of 22 *Cdkl5* -/Y mice were not able to finish 10 trials in 50 minutes, whereas all 22 control mice finished 10 trials in 50 minutes. *Cdkl5* -/Y mice spent significantly longer time to finish 10 trials than control mice (F_1,42_ = 6.92, p = 0.0119, two-way repeated measures ANOVA, Fig 8H). There was no significant difference in distance traveled between genotypes (F_1,42_ = 0.671, p = 0.4174, two-way repeated measures ANOVA, Fig 8I). These results confirmed impaired working memory of *Cdkl5* KO mice.

### Impaired dendritic arborization and immature spine development of hippocampal pyramidal neurons in *Cdkl5* -/Y mice

**Fig 9:**
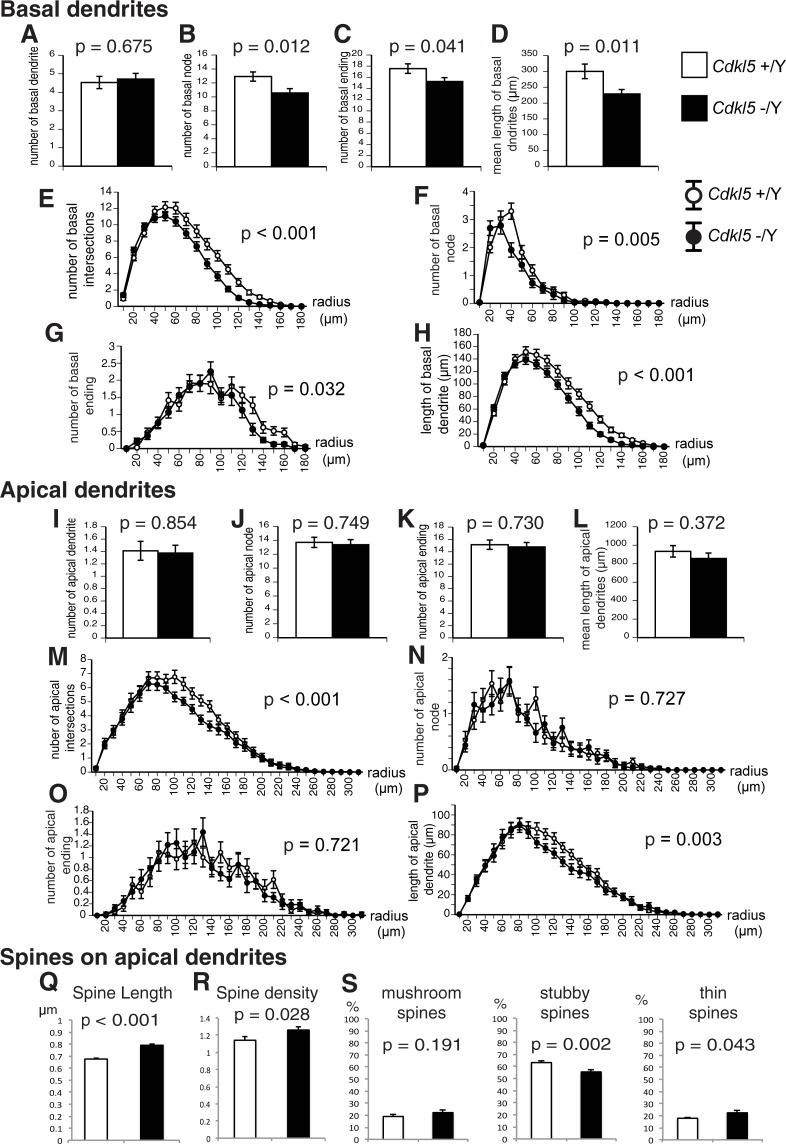
Dendritic arborization and spine geometry of hippocampal CA1 pyramidal neurons are impaired in *Cdkl5* -/Y mice. Results for basal dendrites (A-H) and apical dendrites (I-P). *Cdkl5* +/Y, n = 34 neurons; *Cdkl5* -/Y, n = 32 neurons. Bar graphs (A-D, I-L) show overall mean values, and line graphs (E-H, M-P) show mean values on 10-μm increment concentric circles. There are no significant differences in the mean numbers of basal and apical dendrites between two genotypes (A,I). The number of nodes (B,F), endings (C,G), intersections (E), and length (D,H) of basal dendrites are significantly decreased in *Cdkl5* -/Y mice. The numbers of intersections (M) and length (P) of apical dendrites are significantly decreased in *Cdkl5* -/Y mice. (Q,R) Morphometric analysis of spines on apical dendrites. *Cdkl5* +/Y, 8,150 spines, n = 29 neurons; -/Y, 6,831 spines, n = 32 neurons. The mean length (Q) and density (R) are increased in *Cdkl5* -/Y mice. (S) Spine-type classification analysis. *Cdkl5* +/Y, 3,442 spines, n = 15 neurons; -/Y, 4,294 spines, n = 15 neurons. *Cdkl5* -/Y mice show a significantly lower percentage of stubby spines and a significantly higher percentage of thin spines. (A-D, I-L) are analyzed by ANOVA, (E-H, M-P) are analyzed by repeated measure ANOVA, and (Q-S) are analyzed by Student’s *t*-test. Data indicate mean ± SEM.

We analyzed dendritic arborization and spine geometry of hippocampal neurons of our *Cdkl5* -/Y mice. Amendola et al. previously reported significant reduction in the total dendrite length and the dendritic complexity of pyramidal neurons in the hippocampus of *Cdkl5* exon-4-targeted KO mice [21]. We examined the length and branching of dendrites in hippocampal CA1 pyramidal neurons using the Neurolucida software (S1 Fig). There was no significant difference in the mean number of basal dendrites stemmed from the soma between the genotypes (Fig 9A). However, basal dendrites of *Cdkl5* -/Y mice showed a significant decrease in the mean number of nodes (p = 0.012, Fig 9B), endings (p = 0.041, Fig 9C), and mean length (p = 0.011, Fig 9D) compared to control mice. Sholl analysis with 10-μm increments revealed a significant decrease in the number of intersections (p < 0.001, Fig 9E), number of nodes (p = 0.005, Fig 9F), number of endings (p = 0.032, Fig 9G), and length (p < 0.001, Fig 9H) of basal dendrites in *Cdkl5* -/Y mice. Significant decreases in the number of intersections (p < 0.001, Fig 9M) and length (p = 0.003, Fig 9P) were revealed in the apical dendrites of *Cdkl5* -/Y mice. These results indicate significantly impaired outgrowth and arborization of the dendrites of CA1 pyramidal neurons in *Cdkl5* KO mice.

Recent studies have shown altered spine morphology and synaptic density in cultured CDKL5 knockdown neurons, granule neurons from the dentate gyrus of *Cdkl5* KO mice, CA1 pyramidal neurons of Nex-cKO mice, and in patient iPSC-derived neurons [7, 20, 23]. We crossed the *Cdkl5* KO mouse line with the Thy1-EGFP transgenic mouse line, and examined the density and morphology of dendritic spines in EGFP-positive CA1 pyramidal neurons [33] (S1 Fig). We found significant increases in the spine length (p < 0.001, Fig 9Q) and density (p = 0.028, Fig 9R) of *Cdkl5* -/Y mice compared to the control. *Cdkl5* -/Y mice had a significantly lower percentage of stubby spines (p = 0.002, Fig 9S) and a significantly higher percentage of thin spines (p = 0.043, Fig 9S). These alterations collectively indicate the particular increase of immature, thin spines, which are considered to have impaired signaling capabilities. Thus the loss of CDKL5 leads to impaired dendritic arborization and immature spine development in hippocampal CA1 pyramidal neurons.

## Discussion

Human patients with *CDKL5* mutations are characterized by early-onset intractable epilepsy and severely impaired psychomotor development [4, 34]. As animal models of CDKL5 deficiency, three lines of *Cdkl5* KO mice have been generated by targeting different exons on different backgrounds. To validate an animal model, three criteria are commonly used, i.e. construct validity, face validity, and predictive validity [35]. In the case of *Cdkl5*, the hemizygous male KO mouse models a complete loss of the functional protein. Since most of the human patients are heterozygous female, the KO mouse is not an exact equivalent of the disease model, but it serves as a neurobiological tool to assess the LOF effects of CDKL5 and to find endophenotypes and biomarkers relevant to the human disorder. Regarding the face validity, there are obvious differences in the phenotypes between human patients and KO mice. The most noticeable one relates to the epilepsy. All the reported patients developed severe to moderate epilepsy; however, none of the previously generated KO mice exhibited signs of epilepsy or spontaneous seizures [9, 19, 21]. The reason for this striking difference is unknown, but it should be explained by species difference in epileptogenicity associated with the loss of CDKL5, and possible compensation by functionally redundant proteins in mice. Absence of seizures in the KO mice may hinder elucidating the mechanism of epileptogenesis, but it should aid identification of molecular roles of CDKL5 in physiological functions, owing to the absence of neuronal loss or damage secondarily caused by the excitotoxicity associated with epilepsy. Another phenotypic difference between human patients and KO mice is in motor functions. The psychomotor development is severely impaired in most patients, whereas the motor behaviors of KO mice are relatively spared. The reason for this could also be related to the absence of seizures in the KO mice, which are exempt from the secondary neuronal loss or damage in motor circuits, or to the species difference in the robustness of motor functions upon the loss of CDKL5. Thus, the significance of *Cdkl5* KO mice to the human CDKL5 deficiency disorder may be limited, and the predictive value of the current KO mice has to be evaluated by further investigations. Nevertheless, the behavioral studies of *Cdkl5* KO mice have provided us with a novel insight into the functions of CDKL5 in specific domains of mammalian behaviors. It is critically important to view the behavioral data in a species-specific context and define the phenotypes in each functional domain.

In the current study, we revealed behavioral characteristics of *Cdkl5* KO mice by adopting a battery of comprehensive behavioral tests. Compared with the previous reports of behavioral studies on *Cdkl5* KO mice, our study has two distinguishing features. First, we have covered an unbiased, broader range of behavioral repertoires in locomotion, emotion, sociability, and cognition/memory in one study, in order to obtain a behavioral landscape of the animal, and undetermined features of CDKL5 LOF. Second, whereas previous *Cdkl5* mutant mice were generated on a C57BL/6J background [19–21], our KO mice have a C57BL/6N background, which should exert a different effect on the behavioral responses [36–38]. These original approaches allowed us to obtain more in-depth knowledge on the behavioral consequences of loss of CDKL5.

In accord with other *Cdkl5* mutant mice, we have confirmed significant deficits in multiple learning and memory tasks in our KO mice. These findings strongly support the fundamental and preserved roles of CDKL5 in cognitive function of mammals. The *Cdkl5* KO mice showed impairment in acquisition and long-term retention of spatial reference memory, and in working memory. The spatial reference and working memories are hippocampus-dependent, and long-term retention of spatial reference memory requires interaction between the hippocampus and neocortical regions, such as the entorhinal or perirhinal cortex. Thus the cognitive phenotypes of *Cdkl5* KO mice clearly indicate functional impairment in the hippocampus and its associated circuits. In support of this, we and others have identified functional and morphological alteration in the hippocampal neurons of *Cdkl5* KO mice, such as disruption in AKT/mTOR-S6/GSK-3β signaling pathways, impairment in dendritic arborization and spine development, and defects in neurogenesis and neuron maturation (Fig 9) [20–23]. We have recently shown aberrant and excessive accumulation of postsynaptic GluN2B-containing NMDARs in the hippocampus and enhanced NMDAR-mediated EPSCs and LTP in the CA1 region of *Cdkl5* KO mice [9]. Furthermore, in *Cdkl5* KO mice, GluN2B overaccumulation has been observed in the stratum lucidum of CA3 as well, where GluN2B distribution is almost excluded in wild-type mice [9, 39]. Upregulation of postsynaptic GluN2B has been shown to variously affect cognitive functions. Transgenic mice overexpressing GluN2B under alpha-CaMKII promoter exhibit enhanced LTP and superior ability in learning and memory [40]. In contrast, mice deficient in the insulin receptor substrate protein of 53 kDa (IRSp53) show increased synaptic targeting of GluN2B-containing NMDARs, enhanced LTP, and severe impairment in contextual fear and spatial reference memories [41, 42]. The functional role of LTP is dependent on specific context of neural network, and there are numerous reports of mutant mice that show enhanced LTP and impaired learning and memory [43–47]. It is possible that aberrant and excessive accumulation of GluN2B-containing NMDARs at postsynapses in the hippocampal trisynaptic circuit may interfere with optimal glutamate signaling in *Cdkl5* KO mice. Thus, the loss of CDKL5 alters NMDAR-dependent glutamate signaling, AKT/mTOR-S6/GSK-3β signaling, neurogenesis, and neuronal maturation, all of which should collectively affect functions of the hippocampal formation and impair learning and memory.

Applying the comprehensive assay strategy, we have identified the significant enhancement of anxiety- and fear- related behaviors in *Cdkl5* KO mice for the first time. The KO mice exhibited enhanced anxiety-like behaviors in the light-dark transition, open field, and elevated plus maze tests, and an increased freezing time and a decreased distance traveled during the pretone period in the altered context of contextual and cued fear conditioning test. Multiple brain structures are involved in the regulation of anxiety and fear, such as amygdala, medial prefrontal cortex (mPFC), thalamus, the bed nucleus of stria terminalis, and the hippocampus [48–50]. Although thorough investigation of these regions in *Cdkl5* KO mice have not been conducted yet, above-mentioned data suggest possible involvement of the hippocampus in the etiology of enhanced anxiety and fear. To explain the mechanisms and causal relationship between the enhanced anxiety- and fear-related behaviors of *Cdkl5* KO mice, two hypotheses in opposite directions can be constructed as follows.

One hypothesis is that the functional impairment in the hippocampus-associated circuits causes an enhanced anxiety, which then leads to an increased fear in a novel environment. The hippocampus is a key structure that regulates both cognition and emotion, and the dorsal and ventral subregions are differently involved in these functions. Cytotoxic lesions of the dorsal hippocampus in rodents impair spatial learning but have no effect on anxiety, whereas lesions of the ventral hippocampus reduce anxiety, without effect on spatial memory [48]. Synaptic NMDARs in the ventral hippocampus have been demonstrated to play a key role in anxiety [48]. Conditional KO mice that lack the GluN1 subunit of NMDARs exclusively from the granule cells in the dentate gyrus exhibited dramatically reduced LTP in both the medial and lateral perforant path inputs to the dentate gyrus, and reduced anxiety assessed on the successive alleys test [51]. Moreover, conditional KO mice that lack GluN2B specifically from pyramidal neurons in hippocampal CA1 and granule neurons in the dentate gyrus displayed a reduced anxiety [52]. It is possible that aberrant postsynaptic overaccumulation of GluN2B-containing NMDARs in the hippocampus might render significant anxiogenic property to the mice. Our previous analyses of the hippocampi of *Cdkl5* KO mice have confirmed the postsynaptic overaccumulation of GluN2B in the entire hippocampal formation and the dorsal region by immunoblotting and immunoelectron microscopy respectively, but the ventral hippocampus itself has not been examined. It is necessary to determine the molecular structures and functions of NMDARs in the ventral hippocampus of *Cdkl5* KO mice, in order to identify the altered circuits in cognitive and emotional functions.

The other hypothesis is that the significantly increased freezing and decreased distance traveled in the altered context represent the overgeneralization of fear memories across context, which then leads to the inappropriate anxiety. Overgeneralization of fear is commonly seen in many mental disorders and conditions, such as specific phobia, obsessive-compulsive disorder, panic disorder, generalized anxiety disorder, and posttraumatic stress disorder (PTSD) [53, 54]. Etiologically, overgeneralization of fear arises from the impairment in context discrimination or pattern separation. Failure to discriminate threat from safety should give rise to excessive fear and anxiety. Contextual conditioning and discrimination are regulated by different mechanisms [54–56]. The dorsal hippocampus is essential for context discrimination but not for contextual conditioning, and context discrimination is a more sensitive measure to detect hippocampal dysfunction compared with the conditioning procedure [55]. Recently the neural circuit composed of the mPFC, the nucleus reuniens, and the hippocampus has been identified to control fear memory generalization [56]. The hippocampus also plays a role in discriminating threat from safety through pattern separation, mediated by the dentate gyrus [54, 57]. Therefore, our study indicates that the loss of CDKL5 should affect neural circuits extending from the hippocampus to neocortex that regulate context discrimination or pattern separation. Further investigation of molecular mechanisms of enhanced anxiety and fear by the loss of CDKL5 should aid the elucidation of the pathomechanisms of mental disorders associated with excessive fear and anxiety.

We showed that in contextual and cued fear conditioning test, the freezing time of *Cdkl5* KO mice during conditioning was significantly decreased compared with control mice (Fig 7B). By visual observation, the KO mice seemed to flee in a panic during the conditioning. Previous studies have demonstrated that the degree of the freezing behavior in rodents changes by drugs or genetic manipulation. Pre-administration of d-amphetamine in rats attenuated the freezing and enhanced the avoidance response in fear conditioning [58]. Mutant mice deficient in the 65 kDa isoform of glutamic acid decarboxylase (GAD65) showed reduced freezing and increased flight and escape behaviors compared to their wild-type and heterozygous littermates in contextual and cued fear conditioning, most likely due to deficits in threat estimation or the elicitation of appropriate conditioned fear behavior [59]. Freezing and fleeing are general defensive responses in many species. A discrete and localizable threat source, such as a visible predator, will promote flight, while an ambiguous and difficult-to-locate threat source will promote freezing and risk assessment. Since an animal cannot freeze and flee at the same moment, these defenses are mutually exclusive and therefore controlled by different motor systems [60, 61]. In an experimental model in which either flight or freezing reactions can be elicited by innately aversive ultrasound, mice predominantly displaying freezing had preferential neural activity in the lateral septum ventral and several medial and periventricular hypothalamic nuclei, whereas mice predominantly displaying flight had more activity in the cortical, amygdalar, and striatal motor areas, dorsolateral posterior zone of the hypothalamus, and vertical limb of the diagonal band [62]. Considering that *Cdkl5* KO mice showed robust freezing both one day and seven days after conditioning, it is plausible that a failure to estimate the threat in a novel environment during conditioning may have given rise to an inappropriate tendency toward flight, which could be associated with some functional alteration within above-cited circuits.

It is of particular note that the characteristic anxiety-related phenotypes obtained in this study differ from those in previous reports. The *Cdkl5* exon-6-targeted KO mice have been shown to exhibit hyperactivity, decreased anxiety in a zero-maze assay, and unaltered anxiety in an open field test [19, 27]. The apparent discrepancy in the behavioral data could have resulted from the difference in test protocols used, but we assume that the difference in the background strain could have exerted considerable effect. Previous *Cdkl5* KO mice were generated on the C57BL/6J background, whereas ours were on the C57BL/6N background. C57BL/6J and C57BL/6N substrains were separated from the ancestral C57BL/6 line during 1940s and 1950s, and 0.8% of the SNP loci have been shown genetically distinct between these two substrains [63]. Various differences in behaviors between these two substrains have been identified [36–38]. Matsuo et al. have shown that C57BL/6J is more active and show less anxiety-like behaviors compared to C57BL/6N [37]. Therefore, it is possible that some C57BL/6N-specific factors or modifiers interact with functional alteration induced by the loss of CDKL5 and amplify certain phenotypes such as anxiety-like responses robustly in the KO mice. The strain-dependent modifier effects on anxiety or any other responses can give us a significant insight into the roles and the LOF of CDKL5. Identification of such modifiers should indicate or implicate the specific molecular pathways affected by the loss of CDKL5, and thus facilitate elucidating the interaction targets of CDKL5. Furthermore, the strain-dependent modification of behaviors in *Cdkl5* KO mice can be correlated with the variability of symptoms in patients with *CDKL5* mutations. These findings emphasize the significance and necessity of investigating the behavioral phenotypes of multiple strains and species, in broader context of repertoire, in order to better understand biological effects of CDKL5 LOF, and to find relevant biomarkers applicable to preclinical therapeutic trials to the CDKL5 deficiency disorder.

## Methods

### Ethics statements

All experiments were performed in accordance with the national guidelines and approved by the Animal Experiment Committees of Graduate School of Medicine, the University of Tokyo (P08–073, P13–094) and the Animal Research Committee of the National Institute for Physiological Sciences. For the morphological analysis of neurons, mice were euthanized by cervical dislocation. All efforts were made to minimize suffering of mice.

### Animals and experimental designs

*Cdkl5* KO mice were generated by targeting the exon 2 as previously described [9]. We generated a *Cdkl5* KO mouse line with the C57BL/6N genetic background by repeated backcrosses of selected heterozygous females to C57BL/6N males. Some of the backcrosses were performed by a high-speed congenic method using spermatids from immature males (congenic breeding) [64]. All behavioral tests were carried out with male mice that were 10–11 weeks old at the start of testing. Mice were housed 4 per cage in a room with 12-h light/dark cycles (light on at 7:00 a.m.) with access to food and water *ad libitum*. All behavioral tests were performed between 9:00 a.m. and 6:00 p.m. After all tests, all apparatus was cleaned with diluted sodium hypochlorite water to prevent a bias due to olfactory cues.

We prepared two independent groups of mice for behavioral tests. Experiments were done as follows; the first group of mice (*Cdkl5* +/Y n = 22, *Cdkl5* -/Y n = 22): general health and neurological screening, hot plate test, rotarod test, gait analysis, prepulse inhibition test, light/dark transition test, open field test, elevated plus maze, Porsolt swim test, tail suspension test, one-chamber social interaction test, three-chamber social interaction test, 24-hour social interaction test in home cage, and fear conditioning test; the second group of mice (*Cdkl5* +/Y n = 22, *Cdkl5* -/Y n = 22): Barnes maze test and T-maze test. Each behavioral test was separated from each other by at least 1 day. The raw data of behavioral tests, which are not described in this paper, will be disclosed in the gene-brain-phenotyping database (http://www.mouse-phenotype.org/).

### General health and neurological screening

General health and neurological screen examined the body weight, rectal temperature, whisker, coat, simple reflexes and muscle strength. Grip strength test and wire hang test were used to measure muscle strength. Grip strength was measured by using a grip strength meter (O’Hara & Co., Tokyo) as previously reported [37]. Each mouse was lifted by its tail, and grasped a wire grid by its forepaws. Then, the mouse was gently pulled back by the tail. The peak force applied by forelimbs of the mouse was recorded. In the wire hang test, latency to fall from an inverted wire mesh was recorded.

### Hot plate test

A pain sensitivity was measured by the hot plate test according to the previous report [37]. Each Mouse was placed on a 55°C hot plate (Columbus Instruments, Columbus, OH), and latency to the first hind paw lick was recorded.

### Rotarod test

Motor coordination and motor learning was assessed by the rotarod test [37]. Each mouse was placed on a rotating drum with 3 cm diameter accelerating rotarod (Ugo Basile, Varese), and latency to fall from the rotarod was recorded. The speed of the rotarod accelerated from 4 to 40 r.p.m over a 5-min period. The test was performed three times per day.

### Gait analysis

The gait of each mouse during spontaneous walk/trot locomotion on a translucent plate was shot by a high-speed camera (150 flame/sec.) from underneath. Footprint patterns were analyzed on distance between each stride, variability in stride length, variability around a liner axis, stance width of front paws and hind paws [65, 66].

### Startle response / prepulse inhibition test

For the assessment of sensorimotor gating, a startle reflex measurement system (O’Hara & Co., Tokyo) was used to measure startle response and prepulse inhibition as previously described [37]. A mouse was placed in a plastic cylinder and left undisturbed for 10 min for acclimatization. White noise (40 msec) was used as the stimulus. The intensity of the startle stimulus was 110 or 120 dB, and the prepulse stimulus was presented 100 msec before the startle stimulus, and its intensity was 74 or 78 dB. The startle response was recorded for 140 msec starting with the onset of the prepulse stimulus. A test session consisted of six trial types (i.e., two types for startle stimulus only trials, and four types for prepulse inhibition trials). Four combinations of prepulse and startle stimuli were used (74–110, 78–110, 74–120, and 78–120 dB). Six blocks of the six trial types were presented in pseudorandom order such that each trial type was presented once within a block. The average inter-trial interval was 15 s (range 10–20 s).

### Light/dark transition test

The light/dark transition test was performed as previously described [67]. The apparatus consisted of a cage (21 x 42 x 25 cm) divided into two sections of equal size by a partition with a door (Ohara & Co., Tokyo). One chamber was brightly lit (390 lux), whereas the other chamber was dark (2 lux). A mouse was placed into the dark side and allowed to move freely between the two chambers with the door open for 10 min. The total number of transitions, latency to first enter the light chamber, distance traveled, and time spent in each chamber were recorded by Image LD software (see ‘Image analysis of behavioral tests’).

### Open field test

The open field test was performed as previously described [37, 68]. Each mouse was placed in the corner of the VersaMax open field apparatus (40 x 40 x 30 cm; AccuScan Instruments, Columbus, OH), which was equipped with two layers of infrared light beams traversing the mouse cage from front to back and from left to right. The chamber of the test was lit at 100 lux. The total distance traveled, vertical activity (rearing measured by counting the number of photobeam interruptions), time spent in the center area (20 x 20 cm), and stereotypic counts (the number of times the mouse broke the same beam in succession without breaking an adjacent beam) were recorded for 120 min. Data acquisition was performed automatically using Image OF software (see ‘Image analysis of behavioral tests’).

### Elevated plus maze test

The elevated plus maze test was performed as previously described [68, 69]. The apparatus consisted of two open arms (25 x 5 cm) and two enclosed arms of the same size with 15 cm high transparent walls. The arms and central square were made of white plastic plates and were elevated 55 cm above the floor. Arms of the same type were oriented opposite from each other. Each mouse was placed in the central square of the maze (5 x 5 cm), facing one of the closed arms. Mouse behavior was recorded for 10 min. The number of entries into open arms, and the time spent in the open and enclosed arms were recorded. Percentage of entries into open arms, time spent in open arms, number of total entries, and total distance traveled were analyzed. Data acquisition and analysis were performed automatically using Image EP software (see ‘Image analysis of behavioral tests’).

### Porsolt forced swim test

The Porsolt swim test was performed as previously described [37, 68]. The apparatus consisted of four Plexiglas cylinders (10 cm diameter x 20 cm height). The cylinders were filled with water (23°C) up to a height of 7.5 cm. Mice were placed in the cylinders, and the images were captured at one frame per second for 10 min. Data acquisition and analysis were automatically performed. Immobility time (%) and distance traveled (cm) were measured by Image PS and Image OF software, respectively (see ‘Image analysis of behavioral tests’).

### Tail suspension test

The tail suspension test was performed as previously described [68]. Mice were suspended 30 cm above the floor by adhesive tape placed 1cm from the tip of the tail, and the immobility was recorded for 10 min. Data acquisition was automatically performed using Image TS software (see ‘Data analysis of behavioral tests’).

### Social interaction test in a novel environment (one-chamber social interaction test)

The social interaction test in a novel environment (one-chamber social interaction test) was performed as previously described [70]. Two mice of the same genotypes that were previously housed in different cages were placed in a box (40 × 40 × 30 cm) together and allowed to explore freely for 10 min. Mouse behaviors were recorded with a CCD camera. The total number of contacts, total duration of active contacts, total contact duration, mean duration per contact, and total distance traveled were measured automatically using Image SI software (see ‘Data analysis of behavioral tests’). The active contact was defined as follows. Images were captured at one frame per second, and distance traveled between two successive frames was calculated for each mouse. If the two mice contacted each other and the distance traveled by either mouse was longer than 2 cm, the behavior was considered as 'active contact'.

### Three-chamber social interaction test

The three-chamber social interaction test was performed as previously described [68]. The testing apparatus consisted of a rectangular, three-chambered box and a lid with an infrared video camera (O’hara & Co., Tokyo). Each chamber was 20 cm x 40 cm x 22 cm and the dividing walls were made of clear Plexiglas, with small square openings (5 x 3 cm) allowing access into each chamber. An unfamiliar C57BL/6 male (stranger 1), that had had no prior contact with the subject mouse, was placed in one of the side chambers. The location of stranger 1 in the left vs. right side chamber was systematically alternated between trials. The stranger mouse was enclosed in a small, round wire cage, which allowed nose contact between the bars, but prevented fighting. The cage was 11 cm in height, with a bottom diameter of 9 cm, vertical bars 0.5 cm, and horizontal bars spaced 1 cm apart. The subject mouse was first placed in the middle chamber and allowed to explore the entire social test box for a 10-min session. The amount of time spent in each chamber and number of entries into each chamber was measured. Each mouse was tested in a 10-min session to quantify social preference for the first stranger. After the first 10-min session, a second unfamiliar mouse was placed in the chamber that had been empty during the first 10-min session. This second stranger was also enclosed in an identical small wire cage. The test mouse thus had a choice between the first, already-investigated unfamiliar mouse (stranger 1), and the novel unfamiliar mouse (stranger 2). The amount of time spent in each cage during the second 10-min and number of entries into each cage was measured as described above. Analysis was performed automatically using Image CSI software (see ‘Data analysis of behavioral tests’)

### Home cage monitoring

Twenty-four hour monitoring in the home cage was conducted as previously described [68, 71]. Two mice of the same genotype that had been housed separately were placed together in a home cage (29 x 18 x 12 cm) attached with an infrared video camera. Their social behaviors and locomotor activities were monitored for 1 week. Images from each cage were captured as an 8-bit grayscale at a rate of 1 frame per second. The images were then converted to binary with a threshold level, and processed by filters for noise reduction and shape correction. The final image outlining the continuous contour of a single mouse or contacted mice was designated as a “particle”. Social interaction was measured by counting the number of particles detected in each frame: two particles indicated that the mice were not in contact with each other; and one particle indicated contact between the two mice. Locomotor activity was measured by quantifying the number of pixels that changed between each pair of successive frames. Analysis was performed automatically using Image HA software (see ‘Data analysis of behavioral tests’).

### Contextual and cued fear conditioning test

Fear conditioning tests were performed as previously described [68]. Each mouse was placed in a cuboid chamber (26 x 34 x 29 cm) and allowed to explore freely for 2 min. A 60 dB white noise, which served as the conditioned stimulus (CS), was presented for 30 sec, followed by a mild (2 secs, 0.5 mA) footshock, which served as the unconditioned stimulus (US). Two more CS-US pairings were presented with a 2-min inter-stimulus interval. Context testing was conducted 24 hours and 7 days after conditioning in the same chamber. Cued testing with altered context was conducted after conditioning using a triangular chamber (35 x 35 x 40 cm) made of white opaque Plexiglas. Images were captured at 1 frame per second. For each pair of successive frames, the amount of area (pixels) by which the mouse moved was measured. When this area was below a certain threshold, the behavior was judged as ‘freezing’. When the amount of area equaled or exceeded the threshold, the behavior was considered as ‘non-freezing’. Data acquisition, control of stimuli (i.e. tones and shocks), and data analysis were performed automatically using Image FZ software (see ‘Data analysis of behavioral tests’).

### Barnes maze test

The Barnes maze test was performed as previously described [68, 72]. The maze apparatus consisted of a white circular disk 1.0 m in diameter with 12 holes equally spaced around the perimeter (O’hara & Co., Tokyo). The disk was elevated 75 cm from the floor. A black escape box (17 x 13 x 7 cm), which had paper cage bedding on its bottom, was located under one of the holes, which represented the target. The location of the target was consistent for a given mouse but randomized across mice. The maze was rotated daily, with the spatial location of the target unchanged with respect to the distal visual room cues, to prevent a bias based on olfactory or the proximal cues within the maze. Two to three trials per day were conducted everyday until the total number of trials reached 12. The next day, a probe trial without the escape box (PT1) was conducted to confirm that this spatial task was acquired based on navigation by distal environment room cues. Further 9 trials were given to both genotypes until the KO mice were able to remember the target hole. After 1 month, probe trial tests were conducted to evaluate memory retention. Time spent around each hole was recorded by Image BM software (see ‘Data analysis of behavioral tests’)

### T-maze test

Forced alternation task using the T-maze were performed as previously described with modifications [73, 74]. The apparatus was constructed of white plastics runways with walls 25-cm high (O'Hara & Co., Tokyo) (Fig 8J). The maze was partitioned off into 6 areas by sliding doors. The stem of T was composed of area S2 (13 × 24 cm) and the arms of T were composed of area A1 and A2 (11.5 × 20.5 cm). Area P1 and P2 were the connecting passage way from the arm (area A1 or A2) to the start compartment (area S1). Each trial consisted of one forced run followed by one free run. First the mouse was placed in S2 and forced to proceed in one direction (S2 → A2 → P2 → S1, or S2 → A1 → P1 → S1) by opening the doors successively. After returning to S2, both a1 and a2 doors were made open and the mouse was allowed to choose either direction freely. If the mouse chose the opposite direction from the previous forced run, it was counted as a correct response, and if the mouse chose the same direction as the previous run, it was counted as an incorrect response. Each mouse was given 10 trials per day, for 3 consecutive days. Data acquisition and control of sliding doors were performed automatically using Image TM software (see ‘Data analysis of behavioral tests’), and data were analyzed by two-way repeated measures ANOVA.

### Image analysis of behavioral tests

The applications used for the behavioral studies (Image LD, Image OF, Image EP, Image PS, Image TS, Image SI, Image CSI, Image FZ, Image HA, Image BM, Image TM) were based on the ImageJ program (developed at the U.S. National Institutes of Health and available on the Internet at https://imagej.nih.gov/ij/), which were modified for each test by Tsuyoshi Miyakawa (available through O’hara & Co.).

### Statistical analysis

Statistical analysis was conducted using StatView (SAS Institute, Cary, NC). Data were analyzed by two-tailed t-tests, one-way ANOVA, two-way ANOVA, or two-way repeated-measures ANOVAs. Values in graphs were expressed as mean ± SEM.

### Sholl analysis

*Cdkl5* -/Y and +/Y mice were euthanized by cervical dislocation. Brains were removed and stained using FD Rapid GolgiStain Kit (FD NeuroTechnologies, Columbia, MD) as manufacture’s instruction. Two hundred and forty μm thick coronal slices of Golgi-Cox stained brains were prepared. Hippocampal CA1 regions captured by Leica DM6000 B upright microscope using 40x lens were monitored on computer display and traced using the Neurolucida software (MBF Bioscience, Williston, VT). Concentric circles (Sholl lines) were drawn at 10 μm increments up to a radius of 180 μm from the center of the soma. Following parameters were measured: (1) the size of cell body, (2) number of dendrites, (3) number of nodes, (4) number of endings, (5) total length of dendrites, (6) each length of dendrites, (7) the number of intersection in every 10 μm from the center of cell body, (8) the number of nodes in every 10 μm from the center of cell body, (9) the number of endings in every 10 μm from the center of cell body. All measurements were performed in a blindfolded manner as to genotypes.

### Spine analysis

We crossed the *Cdkl5* KO mouse line with Thy1-EGFP line M transgenic mouse [33]. Thy1-GFP positive *Cdkl5* -/Y and +/Y mice were euthanized by cervical dislocation and transcardially perfused with PBS followed by 4% paraformaldehyde (PFA) in PBS. Brains were removed and drop fixed in 4% PFA for 60 min. Fifty μm thick brain slices were cut using the vibrating microslicer DTK-1000 (D.S.K). Apical dendrites of CA1 pyramidal neurons were imaged by the laser scanning confocal microscope FV1000-D (Olympus, Tokyo, Japan) using 60x oil lens (NA 1.35). Image acquisition was performed with 2048 x 2048 pixel size and 0.1 μm slice interval (Voxel size: 0.1 μm^3^). On the secondary and tertiary branches of CA1 apical dendrites located between 80 and 180 μm from the cell body layers, 6,831 spines from 32 *Cdkl5* -/Y neurons and 8,150 spines from 29 *Cdkl5* +/Y neurons were analyzed using Imaris 7.4 software (Bitplane, Zurich, Switzerland). The Imaris FilamentTracer with AutoDepth Method was used to trace dendrites in Surpass view. After centering filaments, Rebuild Dendrite Diameter was performed. Dendrite Threshold (local contrast) was automatically set. Spine seed point was set to 0.15 μm and maximum length was set to 3 μm. Spine Seed Points Threshold was automatically set and adjusted manually such that all visually discernible spines were detected. Spine Threshold (local contrast) was set at 3. After rendering, we removed the spines that had 0–30 degrees of Spine Orientation Angle from the Z-axis, because the confocal microscopic images had Z-axis blurring, which might lead to errors in automatic rendering. Finally, Dendrite length, Dendrite Area, Dendrite No. of Spines, Dendrite Spine Density, Spine Length, Spine Max Diameter, Spine Mean Diameter, were automatically obtained from the rendered dendrites and spines. Spine types were classified for 3,442 spines from 15 *Cdkl5* +/Y neurons and 4,294 spines from 15 *Cdkl5* -/Y neurons, using NeuronStudio software (Computational Neurobiology and Imaging Center, New York, U.S.A.).

## Supporting information

S1 Fig Representative images of dendrites and spines of *Cdkl5* +/Y and *Cdkl5* -/Y mice. (A) Representative tracings of dendrites of *Cdkl5* +/Y and *Cdkl5* -/Y neurons by Neurolucida. Scale bars = 100 μm. (B,C) Representative images of dendrites from Thy1-EGFP positive CA1 pyramidal neurons. Scale bars = 50 μm (B), and 5 μm (C).
(EPS)
